# Linking primary cilia defects to the “Big 3” neurodegenerative diseases — causal, consequential, or correlative?

**DOI:** 10.17179/excli2026-9499

**Published:** 2026-05-29

**Authors:** Catherine Hong Huan Hor, Bor Luen Tang

**Affiliations:** 1 Department of Chemistry, Faculty of Science, Hong Kong Baptist University, Hong Kong SAR; 2 Department of Biochemistry, Yong Loo Lin School of Medicine, National University Health System, National University of Singapore, Singapore

## ⁯⁯

As a signaling hub harboring neurotransmitter and hormonal signaling receptors, primary cilia defects would be intuitively associated with congenital, early-onset neurological and neuropsychiatric disorders. However, in the past few years, primary cilia defects have also been heavily linked with aging-associated, late-onset neurodegenerative diseases. Are primary cilia defects found in models and patients of Alzheimer's disease, Parkinson's disease and Amyotrophic Lateral Sclerosis causal to disease pathologies and progressions, or are they simply consequences of the latter (or are these merely correlated)?

The primary cilium is a non-motile membranous structure that could be found protruding from the surface of all mammalian cells at some stage of their development. With its shape and morphology rigidified by an underlying microtubule organization, its access is limited by an elaborate selective permeability barrier (the transition zone) and its axonemal content dynamically regulated by complex intraflagellar transport systems, the primary cilium is very much in its own right a subcellular organelle. The axonemal surface has a high concentration of a myriad of signaling receptors, and the primary cilium is thus a unique cell surface signaling hub. The signaling processes and pathways at the primary cilium are universally important, if not critical, for cellular and organismal development, function, and survival. Genetic defects in primary cilia components or factors which regulate cilia biogenesis, maintenance or signaling activities thus resulted in numerous congenital syndromes collectively known as ciliopathies (Mill et al., 2023[8]). These come with a wide range of symptoms affecting multiple tissues and organs, including the brain.

Neuronal cilia harbor receptors for neurotransmitter (such as the 5HTR6 serotonin receptor) and hormonal signaling (such as the somatostatin receptor type 3 (SSTR3) for somatostatin), and primary cilia can form structural functional connections with axons and dendritic spines^1^. As such, cilia defects would be intuitively associated with congenital or early-onset neurological or neuropsychiatric disorders. In the past few years, however, primary cilia structure and function have also been heavily linked with aging-associated, late-onset neurodegenerative diseases, particularly the “Big 3” group of Alzheimer's disease (AD), Parkinson's disease (PD) and Amyotrophic Lateral Sclerosis (ALS). AD, PD, and ALS are the most prevalent causes for aging-related dementia and movement disorder. Despite differences in pathological manifestations, the primary neuronal types affected and the neurological manifestations, all three diseases are characterized by being largely sporadic, and more importantly, late onset in nature. Even the familial forms of these diseases with identified genetic predispositions develop symptoms only years after puberty, as the demise of specific neuronal types progresses beyond a functional threshold. 

Linking congenital ciliary defects directly to the pathology and/or progression of the Big 3 late-onset neurodegenerative diseases could be somewhat non-intuitive because ciliopathy patients typically have a shortened lifespan and neurological symptoms specific to AD, PD or ALS are not usually obvious or readily diagnosed over and above the congenital neurological defects for such patients. How then could we reconcile the above with the growing literature on the association of the primary cilium with AD, PD and ALS? Are the cilia structural or functional defects causal to disease pathologies and progressions, or are they merely consequences of the latter? Alternatively, are these only correlated phenomena, or are there any direct mechanistic links? To better differentiate these possibilities, the cilia-disease links for each of the Big 3 neurodegenerative are examined in turn below.

AD is characterized by the neurotoxicity of various aggregated forms (oligomers, fibrils and plaques) of amyloid beta (Aβ) peptides (generated through amyloidogenic cleavage of Amyloid precursor protein (APP) by BACE1 and the γ-secretase complex), as well as hyperphosphorylated tau (associated with neurofibrillary tangles (NFTs)). Although APP can be found localized to primary cilia, it remains unclear how either of the pathological processes above could be specifically or directly influenced by primary cilia defects. Other than Cyclin-dependent kinase 5 and Tau tubulin kinases 1 and 2, which have known roles in both ciliogenesis and tau phosphorylation, molecular links between cilia function and AD core pathology are sparse. On the other hand, cilia clearly became morphologically altered and functionally defective in AD models (Guo et al., 2025[4]; Huang et al., 2025[5]) and cilia marker alterations have been shown in post-mortem AD brains (Miller et al., 2025[9]). These are due to the disruptiveness and toxicity of Aβ oligomers or aggregates; it thus appears that cilia defects in AD stemming from Aβ are more likely a consequence of AD pathology.

Could cilia defects resulting from AD pathologies in turn contribute to AD pathology or progression? Once again, mechanistic evidence for this notion is not particularly clear. As primary cilia defect would be expected to impair neuroprotective signaling from the organelle, such as those associated with sonic hedgehog (Shh), Wingless-related integration site (Wnt) and ciliary neurotrophic factor, it is conceivable that cilia disruption by Aβ would worsen neuronal survival. Dysregulation of either Shh or Wnt signaling, in particular, has been connected to AD pathogenesis. A recent study demonstrated that inducing defective primary cilia via stereotaxic injection of AAV-Cre into the ventral hippocampus of adult 5xFAD;*IFT88^fl/fl^* mice led to increased Aβ plagues deposition in the septum and ventral hippocampus (Jang et at., 2023[6]). This finding suggests that primary cilium defects may play a role in AD progression. Pending more definitive findings, primary cilia defects in AD could potentially be a causative factor of AD pathology. 

PD is pathologically characterized by dopaminergic neuron demise due to the toxicity of α-synuclein aggregates, a constituent of the histological hallmark of Lewy bodies. Familial PD is associated with many susceptibility gene mutations/variants (*PARK* genes), including α-synuclein (encoded by *SNCA*, or *PARK1*). There are some definitive molecular links between the primary cilia and genetic defects in familial PD, particularly leucine-rich repeat kinase 2 (LRRK2), as activating mutations of which are common underlying causes for familial PD. LRRK2 (*PARK8*) could promote PD pathology through phosphorylation of the small GTPase RAB35, with consequential disruption of endosomal trafficking and lysosomal degradation leading to α-synuclein accumulation and aggregation (Bae et al., 2018[1]). Importantly, LRRK2 is also known to modulate ciliogenesis via its phosphorylation of small Rab GTPases such as RAB8 and RAB10, and loss of primary cilia in specific neuron types is observed in both PD animal models and post-mortem PD brain (Khan et al., 2024[7]). Thus, an LRRK2-activating mutation could parallelly and simultaneously promote both core PD pathology and primary cilia defects. α-synuclein aggregates could also inhibit ciliogenesis, and cilia defects in LRRK2 mutant-induced PD are thus due to a cumulative double-hit by a mutant protein with dual pathological paths. Conceivably, cilia defects and loss of neuroprotective signaling from LRRK2 mutations could further hasten neuronal demise, although it is yet unclear if this would contribute significantly to PD pathology over and above the core mechanisms. 

More recent evidence suggests that another PD susceptibility gene encoding PTEN-induced kinase 1 (PINK1) (PARK6) also has a role in ciliogenesis, and loss of PINK1 also affects both core PD pathology and cilia parallelly and simultaneously, but with mechanisms distinct from that of activated LRRK2 (Bagnoli et al., 2025[2]) (more related to PINK1's role in mitophagy). Therefore, in familial or even sporadic PD, primary cilia defect due to certain genetic mutations or variants (at least for those encoding LRRK2 and PINK1, and perhaps also α-synuclein) could parallelly promote core PD pathology in selectively susceptible neuron types. However, we should realize that these are not the conventional ciliopathy genes (Mill et al., 2023[8]), and their causation of core PD pathology need not go through the primary cilia.

For ALS, a few genes conferring susceptibility to familial forms of the motor neuron disease, such as *C9ORF72*, NIMA (never in mitosis gene a)-related kinase 1 (*NEK1*) and *C21ORF2* have been linked to primary cilia function or defects (Tang et al., 2023[10]; De Decker et al., 2025[3]). *C9ORF72* is particularly interesting, as its intronic GGGGCC repeat expansion resulting in both loss-of-function and gain-of function (toxic expansion transcripts and poly-dipeptides) features underlie about half of the cases of hereditary ALS and frontotemporal dementia. Moreover, motor neurons from patients with *C9ORF72* expansion and *C21ORF2* mutations have cilia defects (De Decker et al., 2025[3]). Together with Smith-Magenis chromosome region 8 (SMCR8), the C9ORF72-SMCR8 complex is a GTPase activating protein (GAP) for Rab8, and this GAP activity negatively regulates ciliogenesis. However, how exactly is the pathological mechanism of mutated *C9ORF72* which leads to motor neuron demise related to primary cilia defect remains unclear. Therefore, although it is likely that pathological features stemming from *C9ORF72 *expansions could cause both neuronal demises and cilia defects parallelly, this it is not yet completely clear. Thus, *C9ORF72* repeat expansion pathology could only be correlated with cilia defects.

In conventional ciliopathies, gene mutations resulting in either defects in primary cilia activity/function, or cilia proteins targeting and association, are usually a principle direct cause of disease pathology. Our brief review of current evidence for the Big 3 however suggests that in none of these neurodegenerative diseases is the observed primary cilia defect a primary cause of core disease pathology. For AD, primary cilia defects are likely both a cause and a consequence of disease progression. In PD, certain genes mutations can act parallelly to promote both PD pathology and cilia defect simultaneously, but the former is not dependent on the latter. For ALS, pending further investigations, motor neuron pathology and primary cilia defects are at the moment only correlative phenomena. While cilia defects induced by the core pathological mechanisms of AD, PD and ALS could in turn negatively impact disease progression, such impacts would be secondary. 

Recognizing the difference between the primary causal impact of cilia defect or dysfunction in congenital or early-onset neurological diseases as opposed to late-onset neurodegenerative disorders is important because it would influence our choices of therapeutic targets and approaches. For ciliopathies, strategies that preserve or promote primary cilia integrity would be useful. However, the same approaches might not be equally impactful for AD, PD, and ALS.

## Notes

Catherine Hong Huan Hor and Bor Luen Tang (Department of Biochemistry, Yong Loo Lin School of Medicine, National University Health System, National University of Singapore, Singapore; bchtbl@nus.edu.sg) contributed equally as corresponding author.

## Declaration

### Conflict of interest

The authors declare no conflict of interest. 

### Artificial Intelligence (AI) - assisted technology

No artificial intelligence program was used to assist in writing any part of the manuscript.
